# Computational Psychometrics Meets Hollywood: The Complexity in Emotional Storytelling

**DOI:** 10.3389/fpsyg.2016.01753

**Published:** 2016-11-08

**Authors:** Pietro Cipresso, Giuseppe Riva

**Affiliations:** ^1^Applied Technology for Neuro-Psychology Laboratory, Istituto Auxologico ItalianoMilano, Italy; ^2^Department of Psychology, Catholic University of MilanMilano, Italy

**Keywords:** modeling, computational psychometrics, communication, psychometrics, storytelling, emotions, media psychology

## Abstract

Expressions of emotions are pervasive in media, especially in movies. In this article, we focus on the emotional relationships of movie characters in narrative thought and emotional storytelling. Several studies examine emotion elicitation through movies, but there is a gap in scientific literature and in the practice to quantitatively consider emotions among the characters of a movie story, which in turn provide the basis of spectator emotion elicitation. Some might argument that the ultimate purpose of a movie is to elicit emotions in the viewers; however, we are highlighting that the path to emotional stimulation entails emotions among the characters composing a narrative and manipulating to enable the effective elicitation of viewers' emotions. Here we provided and tested an effective quantitative method for analyzing these relationships in emotional networks, which allow for a clear understanding of the effects of story changes on movie perceptions and pleasantness.

## Introduction

Many special effects are used to visualize a fantasy which can simply be imagined. So why take this action? The relationship between cinema arts and psychology is very close and this can be easily understood by referring to the relationship between the sense (such as the visual one) and our internal representation of what is shown. However, this wonderful fifth art, cinema, is much more than this relationship. Watching a movie is more than mere perception; this involves de facto sensations, motivations, emotions, and many other aspects of the sphere of human psychology (Gallafent, 2014; Tarnay, 2016). Moreover, the sense of being within a movie or narrative makes the comprehension of this art special and complex (Simons, 2008; Guha et al., 2015). Professional movie makers have provided exemplary real pieces of art which are capable of eliciting much more emotions than real-life experiences (Tarnay, 2016). The narrative is one instrument that is able to turn a simple movie into a grand story. This is one of the most powerful techniques to induce emotions, and it stimulates our imaginations and evokes both familiar personal experiences and new ones. Characters in a movie are able to elicit emotions in us, but how are their emotions in the viewer's perception? Characters' emotions are important because the entire movie narrative depends on the expressed and received emotions. A history narrated in a movie can completely change by expressing the emotions experienced and transmitted by the characters in a different way (Liu et al., 2013).

Several studies examine users' experience during media (Riva et al., 2003; Bryant and Oliver, 2009; Nabi and Oliver, 2009; Chirico et al., 2015; Tarnay, 2016); however, there is no research, to the best of our knowledge, on the emotions of the characters in a movie. Characters and the relationships between them are the backbone of a narrative, and they need to be accurately considered in all aspects. Emotions in particular are peculiar behaviors which significantly affect the entire story. Previous studies have analyzed the networks among characters based only on information like marriages and economic relationships with the purpose of providing a wider protorealistic sense of the narrated universe (Trabasso and van den Broek, 1985; Bearman and Stovel, 2000; Newman and Girvan, 2004; Mason and Thomas, 2007). Also, this technique has been used widely throughout history (Jackson, 2008). For example, Padget studied the economic relationships and marriages of the de Medici family during the Italian Renaissance (Padgett and Ansell, 1993; Padgett, 2011). This approach, however, was based on objective data because the purpose was to understand the de Medici family power based on several types of relationships they forged. In studying movies, the purposes are different. In particular, we are interested in the following: (1) the impact of expressed emotions on the viewers, and (2) storytelling, which is in the movie maker's construction of the story and its expressed emotions.

Several emotions can be elicited during a movie's view (Aurier and Guintcheva, 2015; Gabert-Quillen et al., 2015; Uhrig et al., 2016). There are four emotions that are largely elicited in most cases: anger, fear, sadness, and joy (Philippot, 1993; Rainville et al., 2006; Plutchik and Kellerman, 2013; Buck, 2014). These emotions are considered to be an automatic response to a stimulus (Rainville et al., 2006). However, the emotional processes are more complex than simple reactions (Buck, 2014). As argued by LeDoux, when a visual elicitor is present, its representation reaches the sensory thalamus and from there follows two parallel roads: (1) Down a quick and dirty (low) road, the stimulus directly reaches the amygdala. (2) Down a slow but accurate road, the stimulus reaches the amygdala by passing through the sensory cortex, which is able to further elaborate the content of the stimulus for a better (albeit slower) perception of the stimulus (Ledoux, 1986; LeDoux, 2012a,b; LeDoux et al., 2014).

Studies of emotions need to consider another aspect essential to movie perception—context. The same sequence may have different emotional significances. For example, depending on the context within which it is expressed, a smile from one character to another may express joy as well as anger (Davidson et al., 2000; Buck, 2014).

The context within which the emotions are expressed and perceived has been demonstrated being more important than the facial expressions. In particular Aviezer et al. (2008) reported in their article, three studies demonstrating that “identical facial configurations convey strikingly different emotions and dimensional values depending on the affective context in which they are embedded.” Facial expressions need to be integrated to the situations in which the actions are expressed and depends on several factors, such as the environment and the narrative dimension within which a specific emotion occurs (Dixon-Gordon et al., 2015; Schmader and Mendes, 2015). This is one of the reason for which automatic recognitions of emotions by computers is so complex and maybe impossible, while it is so simple for humans, that are able to understand the narrative context identifying the correct emotions during an action.

The aim of this article was to provide a method to analyze emotions in movies and novels by using the perceived emotional level of the characters.

At individual level a character can be seen as a unit that can “receive” (input) or “send” (output) a behavior. In general term, if a relationship between two nodes in a network has no direction (such as friendship or marriage), then there is no input or output and each node is simply connected to others, and the network will be called undirected. Otherwise if the relationship has a direction (such as the network based on email communication from a node to another) than the network is called directed. Another aspect to consider is the weight of the relationship. When each edge is equivalent in the network, it is called unweighted, otherwise it will be possible to give a specific weight to each edge and the network will be called weighted. A weight can be either the number of times that a relationship occurs (for example the number of emails received give a “weighted-in” relationship and the number of emails sent give a “weighted-out” relationship) or a specific weight, that is defined a priori, representing the strength of each relationship.

We attempted to heavily use weighted directed networks for the analysis. For example, in a network perspective, a smile from “Pietro” to “Giuseppe” could be included in the joy network and/or the anger one. Moreover, the same relationship could be included in the opposite direction, from “Giuseppe” to “Pietro” again in both networks. Complex networks have the power to build structured information from the bottom up; they are built defining low-level relationships to infer social behavior and information which without a network can be observed, only without a clear structure. Once built, a network provides information thanks to synthetic indices per node within the network. In particular, degree indices, in one emotion network, represent how much emotion is expressed (weighted outdegree) or received (weighted indegree) from each character in that emotion network (Newman, 2001; Barrat et al., 2004; Opsahl et al., 2010; Cipresso, 2015). The degree represents the number of links that involve a node.

$$\begin{array}{l} d_{i}\left(g\right)=\#\left\{j:g_{ji}=1\right\}=N_{i}\left(g\right). \end{array}$$

When considering directed network the above formula is for the in-degree index. For the out-degree we need to consider the different path. The weighted degree formulation consider weights in all the paths. Alternatively we can consider the weights for the in indegree or outdegree paths, obtaining the relative indexes.

## Experimental study

To pilot test the idea that we presented, we selected 11 participants to watch and judge the emotional patterns of the characters of a movie. The movie chosen was *Pulp Fiction* by Quentin Tarantino (Gallafent, 2014). The main characteristic of this movie pertinent to our analysis is its nonlinear narrative dense of emotions in the characters. The structure of this movie makes it more difficult than others to develop a clear picture of each character and the movie as a whole (Simons, 2008; Gallafent, 2014).

### Procedure

Four matrices were prepared by the researchers for the movie characters' emotional evaluations. Each matrix was structured with the characters' names repeated in both the first row and the first column. Each participant was asked to watch the movie with as many interruptions as needed. Also, participants were able to go back and forth to make any revisions or to detail their choice for the four emotions. The task for the participants was to fulfill four matrices, one per emotion, with the insertion of a Likert value (from 1 to 10) in the matrices' cells. For example, the cell corresponding to the character “Vincent Vega” in the row crossing the character “Butch Coolidge” in the column in the fear matrix means “fear from …to …” from “Vincent Vega” to “Butch Coolidge.” The interactions are directional in this way. Since the row corresponds to the sent emotion (“from”) and the column corresponds to the received emotion (“to”), it is possible to have different values in the cells, even for the symmetric values with respect to the diagonal. Fear from “Vincent Vega” to “Butch Coolidge” can be entirely different with respect to fear from “Butch Coolidge” to “Vincent Vega.” Participants were able to fill as many cells as needed. The overwriting values were added to the upcoming, so that if an emotional interaction between two characters was repeated, then the new interaction made that link stronger than before with a higher weight for the link.

At the end of this procedure, we used a measure of disagreement denoted “information-based measure of disagreement” (IBMD) for more than two observers (Costa-Santos et al., 2010; Henriques et al., 2013) based on Shannon's notion of entropy (Shannon, 1996). This is defined as the average amount of information contained in a variable, a tool to compare the degree of observer disagreement, and with a 95% confidence interval. This coefficient equals 0 when the observers agree or when there is no disagreement, and it reaches 1 when the distance, the disagreement among the observers, increases. We considered in the matrices only the values with an upper bound of the 95% confidence interval lower than 0.15. A higher level for this parameter would provide more data to populate the four networks, but it would be based on a higher level of disagreement among the 11 judges and could provide inaccurate conclusions. Finally, intraclass correlation coefficient (ICC) was also used to compute the agreement for any couple of the 11 judges, in order to verify any possible divergences respect to the IBMD index.

With this procedure, we built four directional weighted networks for a better understanding of emotional storytelling for the selected movie. The use of the ICC and IBMD indexed provided a warranty of agreement in the emotional evaluations.

## Results

Table 1 displays an extract of the top 10 characters per each emotion, with respect to the weighted indegree and weighted outdegree centrality parameters (Opsahl et al., 2010). Table 2 displays the descriptive statistics for the considered parameters.

**Table 1:** **Weighted indegree and weighted outdegree parameters for the top ten characters in each emotional network (anger, fear, joy, and sadness)**.

**Anger**				**Fear**			
**Id**	**Weighted indegree**	**Id**	**Weighted outdegree**	**Id**	**Weighted indegree**	**Id**	**Weighted outdegree**
Vincent Vega	77	Vincent Vega	96	Butch Coolidge	43	Vincent Vega	62
Butch Coolidge	50	Jules Winnfield	79	Jules Winnfield	41	Butch Coolidge	41
Jules Winnfield	50	Butch Coolidge	54	Vincent Vega	40	Marsellus Wallace	25
Lance	34	Marsellus Wallace	39	Mia Wallace	29	Jules Winnfield	23
Jody	33	Lance	23	Marsellus Wallace	25	Mia Wallace	20
Marsellus Wallace	23	Honey Bunny (Yolanda)	20	Lance	20	Honey Bunny (Yolanda)	20
Fabienne	20	Fabienne	18	Brett	18	Brett	15
Others	19	Pumpkin (Ringo)	17	Others	16	Fabienne	11
Pumpkin (Ringo)	19	Mia Wallace	14	Fabienne	15	Diners	11
Brett	12	Jody	12	Pumpkin (Ringo)	12	Pumpkin (Ringo)	10
**Joy**				**Sadness**			
**Id**	**Weighted indegree**	**Id**	**Weighted outdegree**	**Id**	**Weighted indegree**	**Id**	**Weighted outdegree**
Vincent Vega	65	Vincent Vega	86	Butch Coolidge	14	Butch Coolidge	12
Mia Wallace	39	Butch Coolidge	52	Vincent Vega	12	Fabienne	12
Butch Coolidge	30	Jules Winnfield	42	Fabienne	7	Mia Wallace	8
Jules Winnfield	29	Mia Wallace	33	Others	3	Jules Winnfield	6
Fabienne	28	Fabienne	19	The Gold Watch	3	Vincent Vega	4
Others	21	Honey Bunny (Yolanda)	18	Jules Winnfield	2	Drug	2
Drug	18	Pumpkin (Ringo)	15	Mia Wallace	2	Marsellus Wallace	2
Marsellus Wallace	16	Lance	12	Captain Koons	1	Captain Koons	1
Pumpkin (Ringo)	16	Jimmie Dimmick	10	Drug	1	Honey Bunny (Yolanda)	1
Esmarelda Villalobos	11	The Wolf (Winston)	9	Lance	1	Others	0

**Table 2:** **Weighted indegree and weighted outdegree parameters' descriptive statistics**.

	**Weighted indegree**	**Weighted outdegree**	**Weighted degree**
Mean	8.235	8.235	16.47
Std. Error of Mean	1.170	1.403	2.508
Median	2.000	2.000	4.500
Std. Deviation	13.65	16.37	29.25
Maximum	77.00	96.00	173.00

**Figure 1:**
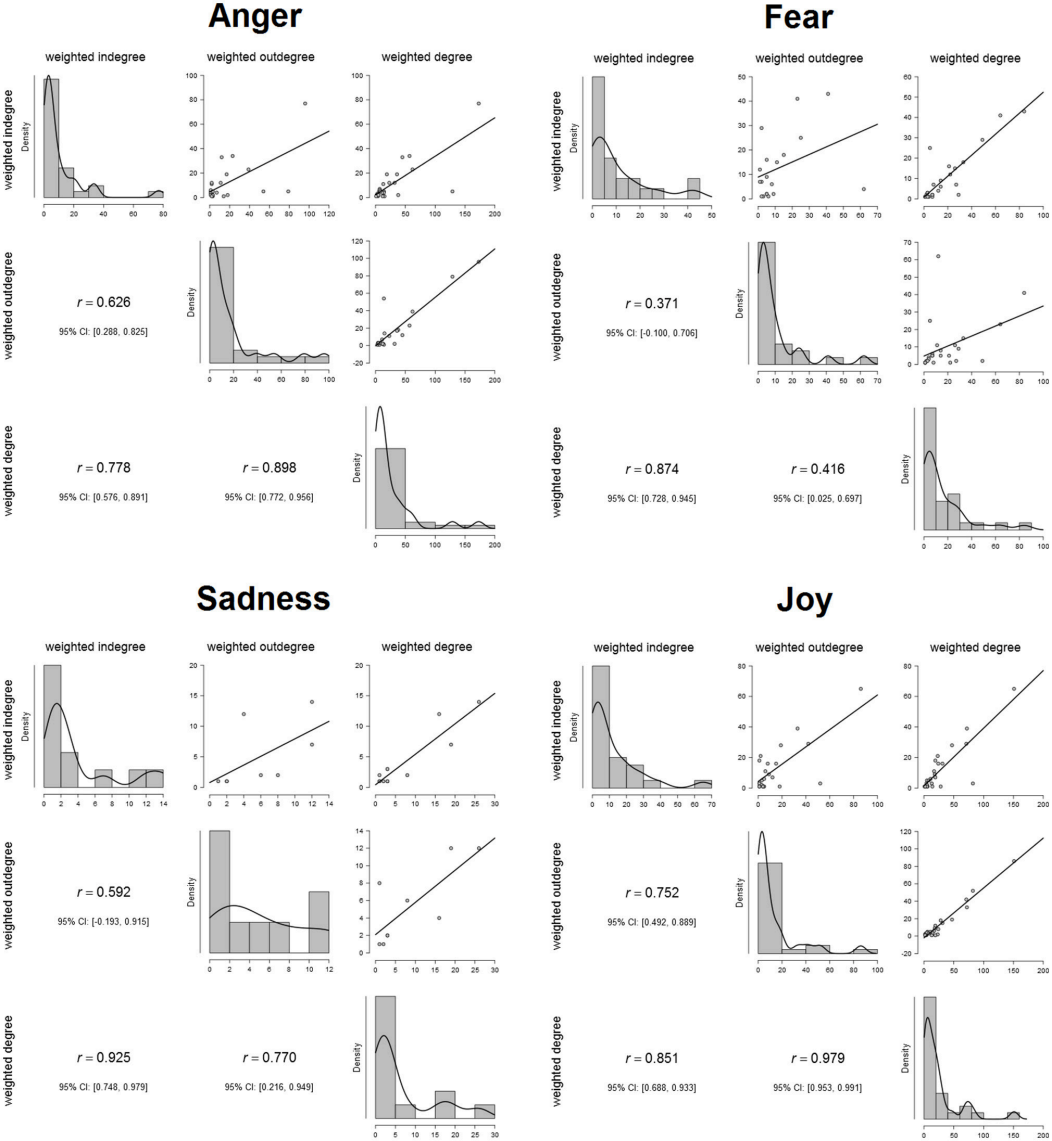
**Relationships among the degree parameters of each emotion**.

The relationships between outdegree and indegree parameters are really strict, which demonstrates a very strong correlation between the measures of each emotion (Figure 1). These results indicate that, according to this analysis, there is a general equilibrium between the expressed emotions from the characters and the received ones.

**Figure 2:**
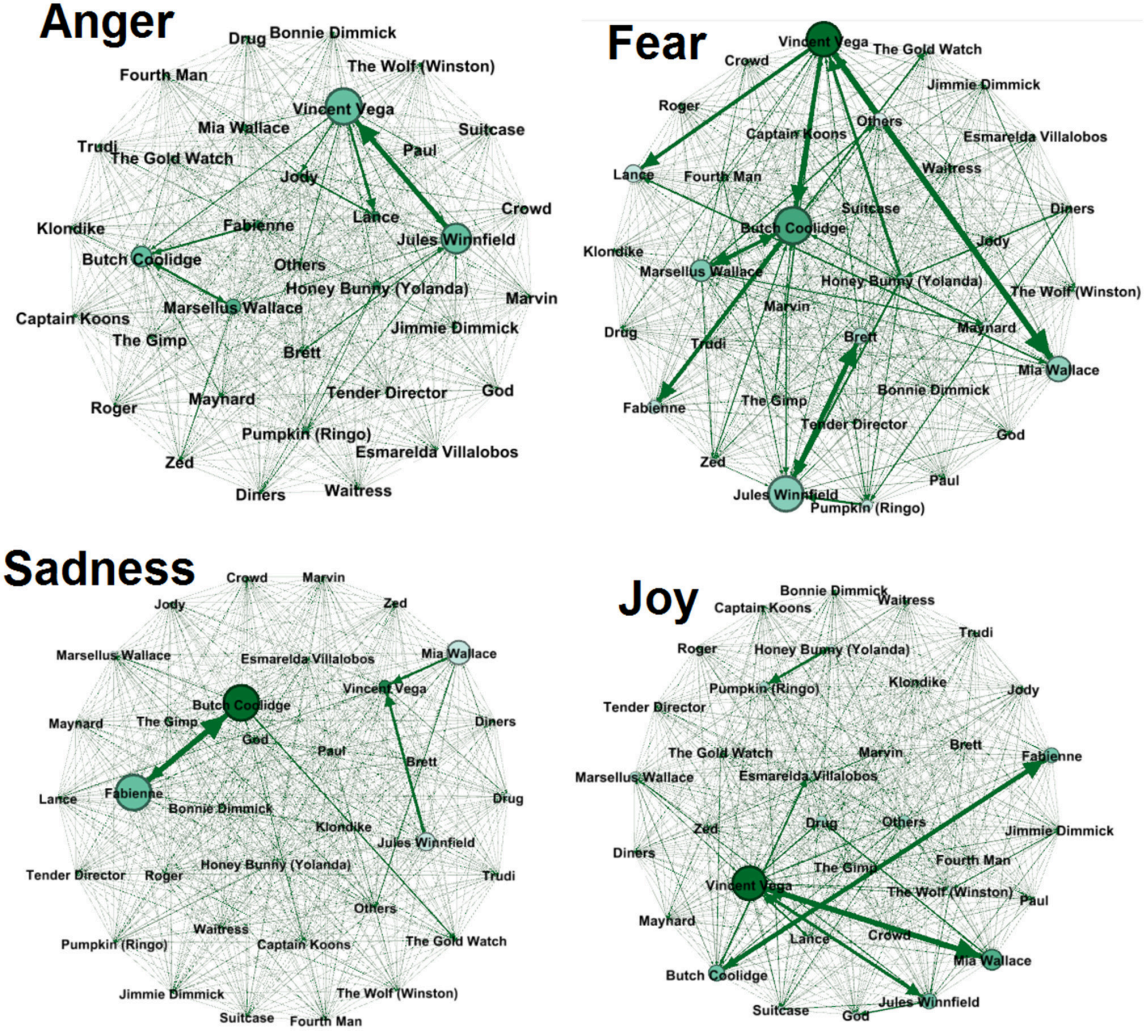
**Graphical representation of the four emotion networks**.

The four networks, one per emotion, are graphically represented in Figure 2. Nodes Color darkness represents the weighted outdegree and nodes dimension represents the weighted indegree. The dimension of the link between two nodes represents the weight of the tie. The arrow represents the direction of the emotion (from one node to another), and so identifies the sender and the receiver per emotion. The network graphs provide an effective way to display results and to inform about the impact of characters and their emotions in the narrative.

## Conclusions

In this article, we demonstrated an innovative technique to study emotions as perceived by viewers in relation to the characters in a movie. Our experimental study showed the potential of this method and a new perspective in the psychology of media and emotions by using computational psychometrics in media (Riva, 2002; Cipresso et al., 2015a,b). However, our article also represents a practical way to infer emotional information during emotional storytelling and in building the screenplay. So, might Quentin Tarantino make better films with these methods and analyses? We do not think this is the case. However, having proper access to emotional information provides additional tools to highlight inside experiences in an artist (Bourgeois-Bougrine et al., 2014). We can infer that wider knowledge can only further open an enlightened mind, like Tarantino.

The method presented here also suggests possible tools for the media industry. Creating an effective emotional narrative is difficult, and this tool may significantly help in defining the right characters within the narrative. The Hollywood movie industry, at all levels, should use these types of tools for creating more effective and emotional narratives, also in tandem with the many consultants who are hired to help in creating the final product. Often, preliminary versions of movies are shown to test audiences to glean information about the general favorability of the movie (d'Astous and Colbert, 2002; d'Astous et al., 2005; Wang et al., 2010). The use of a sample of moviegoers can be even more in-depth than this by including physiological measures and the type of analysis presented here. The particular method we presented can be the first step in understanding the emotional universe, that has been demonstrated to be crucial in screenplay writing (Field, 2007; Hauge, 2011; Bourgeois-Bougrine et al., 2014). At the present time, the perceived emotions of viewers are taken into account, but viewers' perceptions of the emotions expressed by characters have never been considered, but this may constitute an integral part of emotional storytelling and arts production.

## Author contributions

PC and GR conceived the idea for this research. PC and GR analyzed the statistical data. PC conducted the computational analyses and wrote the first draft of the article. PC and GR revised and approved the last version of the article.

### Conflict of interest statement

The authors declare that the research was conducted in the absence of any commercial or financial relationships that could be construed as a potential conflict of interest.
